# Case Study on Incentive Mechanism of Energy Efficiency Retrofit in Coal-Fueled Power Plant in China

**DOI:** 10.1100/2012/841636

**Published:** 2012-12-16

**Authors:** Donghai Yuan, Xujing Guo, Yuan Cao, Liansheng He, Jinggang Wang, Beidou Xi, Junqi Li, Wenlin Ma, Mingshun Zhang

**Affiliations:** ^1^Key Laboratory of Urban Stormwater System and Water Environment, Ministry of Education, Beijing University of Civil Engineering and Architecture, Beijing 100044, China; ^2^Beijing Climate Change Response Research and Education Center, Beijing University of Civil Engineering and Architecture, Beijing 100044, China; ^3^Key Laboratory of Development and Application of Rural Renewable Energy, Biogas Scientific Research Institute of the Ministry of Agriculture, Chengdu 610041, China; ^4^Water Environment System Project Laboratory, Chinese Research Academy of Environmental Sciences, Beijing 100012, China; ^5^Department of Environmental Science and Engineering, Beijing University of Chemical Technology, Beijing 100029, China

## Abstract

An ordinary steam turbine retrofit project is selected as a case study; through the retrofit, the project activities will generate emission reductions within the power grid for about 92,463 tCO_2_e per annum. The internal rate of return (IRR) of the project is only −0.41% without the revenue of carbon credits, for example, CERs, which is much lower than the benchmark value of 8%. Only when the unit price of carbon credit reaches 125 CNY/tCO_2_, the IRR could reach the benchmark and an effective carbon tax needs to increase the price of carbon to 243 CNY/tce in order to make the project financially feasible. Design of incentive mechanism will help these low efficiency enterprises improve efficiency and reduce CO_2_ emissions, which can provide the power plants sufficient incentive to implement energy efficiency retrofit project in existing coal-fuel power generation-units, and we hope it will make a good demonstration for the other low efficiency coal-fueled power generation units in China.

## 1. Introduction

With the rapid development of industrialization and urbanization, the global climate-change issue has become an important factor to affect the world economic order, political regime and international relations, as well as to determine the key of the world's energy future [1, 2]. Global climate change is closely related to energy, a variety of greenhouse gases (GHG) that cause climate change. Carbon dioxide (CO_2_) contribution rate is more than 50%, and 70% of human activities' CO_2_ emissions are from the burning of fossil fuels [3]. At present, China is one of the countries with the most CO_2_ emissions in the word, and CO_2_ emissions are still growing rapidly. The main reasons for CO_2_ emissions of China are as follow: (1) The existing energy resource characteristics of primary energy consumption are dominated by coal, and thermal power accounts for about 70% of the total power generation; (2) the rate of energy-intensive industries and products is high [4].

In the 12th Five-Year Plan, China has shown its intention to shift from a policy of maximizing growth to balance growth with social harmony and environmental sustainability [5]. But the dominant position of coal in the energy consumption will continue in a long period; by 2020, the GDP goal is quadrupling, and then the total installed capacity will reach 900–950 million kilowatts, generating capacity will reach 4.2 trillion kWh, of which thermal power installed capacity still contains about 70%. How to coordinate the relationship between the rapid development with CO_2_ emissions is a severe challenge for the power industry.

Today, there are lots of low efficiency power plants in China, which is urgent to implement the steam turbine retrofit, and these power plants have the great potential to reduce CO_2_ emissions. Because the development among different regions and industries in China is very uneven, the enterprises cannot afford the high cost of the steam turbine retrofit by themselves [6]. The introduction of an incentive mechanism to help these low efficiency enterprises to improve efficiency and reduce CO_2_ emissions has been practically significant [7]. In this study, a steam turbine retrofit project in China is selected for a case study to find an effective GHG emission reduction mechanism for this project type, which can provide the power plants sufficient incentive to implement energy efficiency retrofit project in existing coal fuel-power generation-units, and we want to make a good demonstration for the other low efficiency coal-fuel power generation-units in China.

## 2. Description of the Project Activity

### 2.1. Site Description

**Figure 1:**
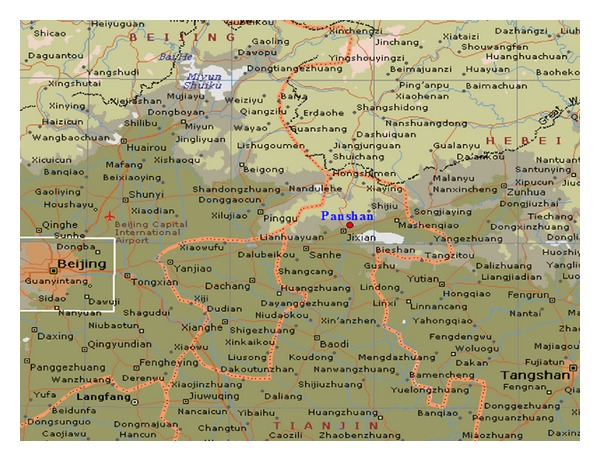
The map of the project location–Panshan Power Plant.

Panshan Power Plant is located in the southeast of the Ji County, Tianjin City (117°16′58′′E, 39°59′26′′N). The mean annual temperature in Ji County is 10-11°C, and the mean annual precipitation is approximately 700 mm, of which three-quarters are distributed from July to September.  Figure 1 is the location of Panshan Power Plant. 

### 2.2. Description of the Project

Steam Turbine Retrofit Project of Tianjin Panshan Power Plant (hereafter refers to as the project) involves retrofitting supercritical steam turbine. The steam turbine with rated power of 500 MW (hereafter refers to as PAT, project activity turbine), which was designed in the early 1970s and introduced from Russia, has been put into commercial operation since April 16th 1996. The technical lifetime of the power unit is 24 years. Designed as a super critical turbine set, the technological level of the PAT is relatively higher than the subcritical turbine sets that are commonly used and regarded as a good practice currently in current China. So up to 2009 (the year of retrofit action), the remaining life is 11 years, more than 8 years. The principal specifications of the steam turbine are shown below (Table 1).

**Figure 2:**
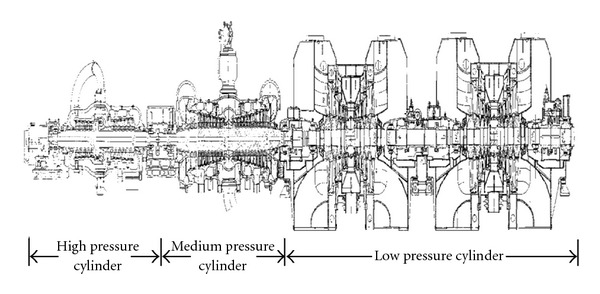
Profile of turbine.

The main target of the project is retrofitting the low pressure cylinder to reduce the coal consumption of the power generation, in particular, by promoting the performance of the low pressure cylinder. The components, including rotor, blades, diaphragm and its set, inner cylinder, and shaft butt seal of the low pressure cylinder, are retrofitted. Steam seal installed in the surrounding bend of the first stage of high-pressure cylinder, steam seal of each turbine stage, and shaft butt seal of high-cylinder and medium-cylinder will also be altered (Figure 2). 

The current practice with low efficiency would be continued in the absence of the proposed project. By adopting retrofit measures, the proposed project will not only reduce GHG emissions, but also contributes to sustainable development for local communities by the means of: (1) reducing the emissions of SO_2_, NO_*x*_, and coal ash due to the reduction of standard coal consumption; (2) improving the energy efficiency of power plant and promoting development of manufacturing industry. The project is expected to give a lead on turbine retrofit of supercritical power plants in China.

## 3. Performance Assessment 

### 3.1. Emission Reductions

To evaluate the effect of GHG emission reduction by the project with a quantitative way, the approved CDM methodology AM0062 in Unit Nation Framework Convention on Climate Change (UNFCCC) is applied to this study on the project in the following steps: calculate the baseline emissions, the project emissions, and the emission reductions [8].

#### 3.1.1. Calculate the Baseline Emissions

(1) Determine baseline emission for the scenario of project electricity generation. Electricity generation in the project power plant will displace in the baseline scenario less efficient electricity generation in the project plant and can, in addition, displace electricity to the grid, if the quantity of electricity generation is increased as a result of the project. The calculation of baseline emissions is therefore based on different emission factors for different quantities of electricity generated. In China the annual power generation is determined by the annual dispatch order from grid company where the PAT connects to, thus it is assumed that annual power generation dispatch will be according to the original capacity of PAT. In this case, the annual power generation after retrofit unlikely exceeds the historical average level. Therefore the emission reduction is only relevant to the efficiencies of PAT before and after the retrofit. 

To evaluate the emission reduction due to the retrofit, the follow case from AM0062 [9] is selected in a future analysis: the quantity of electricity generated in the project turbine (EG_PJ,y_) is lower or the same as the historic average annual generation level (EG_AVR_). Baseline emissions are calculated as:

(1) BEy=EGPJ,y∗EFBL,y,

where BE_y_ = baseline emissions in year “y” (tCO_2_/yr), EG_PJ,y_ = quantity of electricity supplied by the project turbine to the grid in year “y” (MWh/yr), adjusted for changes in efficiency, EF_BL,y_ = baseline emission factor of the project turbine in year y (tCO_2_/MWh).

(2) Determine Baseline Emission factor. The project turbine is steam turbine and the fuel is fired in a boiler, so its emission factor is calculated as follows [10]:

(2) EFBL,y=3.61000×EFFF,BL×FCPJ,y×NCVFF,PJηBL,y×HIPJ,y,

where EF_BL,y_ = baseline emission factor of the Project turbine in year “y” (tCO_2_/MWh), EF_FF,BL_ = CO_2_ emission factor of the fossil fuel used in the Project turbine prior to the implementation of the Project (tCO_2_/TJ), NCV_FF,PJ_ = net calorific value (NCV) of fossil fuel used in the Project turbine during year y (TJ/tonne of fuel).

*η*_BL,y_ = energy efficiency of the turbine without retrofitting estimated using the latest version of approved “tool to determine the baseline efficiency of thermal or electric energy generation systems,” determined the efficiency based on measurements and used a conservative value, based on performance tests before the implementing the project following national/international standards, at discrete loads within the operating range or over the entire rated capacity, FC_PJ,y_ = Actual fuel consumption by project in year “y” (tonne of fuel), HI_PJ,y_ = Heat input to the steam turbine in year “y” (TJ). In case of multicylinder steam turbines, this is the sum of the heat input at the inlet of first stage and the heat inputs in the re-heaters of steam between various cylinders (e.g., high-pressure, medium pressure, and low-pressure cylinders).

#### 3.1.2. Calculate the Project Emissions

The CO_2_ emissions from fossil fuel consumption in the project (PE_y_) should be calculated using the latest approved version of the *“*tool to calculate project or leakage CO_2_ emissions from fossil fuel combustion,” where the process j in the tool corresponds to the combustion of fossil fuels in the project for electricity generation in the project power plants [11]:

(3) PEFC,j,y=∑iFCi,j,y×COEFi,y,

where PE_FC,j,y_ = are the CO_2_ emissions from fossil fuel combustion in process j during the year y, FC_i,j,y_ = is the quantity of fuel type i combusted in process j during the year y (mass or volume unit/yr), COEF_i,y_ = is the CO_2_ emission coefficient of fuel type i in year y (tCO_2_/mass or volume unit), i = are the fuel types combusted in process j during the year y. 

The CO_2_ emission coefficient COEF_i,y  _ can be calculated using one of two options proposed in the “tool to calculate project or leakage CO_2_ emissions from fossil fuel combustion”.Depending on the availability of data on the fossil fuel type i, the CO_2_ emission coefficient COEF_i,y_ is calculated based on net calorific value and CO_2_ emission factor of the fuel type i:

(4) COEFi,y=NCVi,y×EFCO2,i,y

where COEF_i,y_ = is the CO_2_ emission coefficient of fuel type i in year y (tCO_2_/mass or volume unit), NCV_i,y_ = is the weighted average net calorific value of the fuel type i in year y (GJ/mass or volume unit). EF_CO_2_,i,y_ = is the weighted average CO_2_ emission factor of fuel type i in year y (tCO_2_/GJ), i = are the fuel types combusted in process j during the year y. 

#### 3.1.3. Calculate the Emission Reductions

(1) Emission reductions are calculated as follows:

(5) $$\begin{array}{c} \text{E}{\text{R}}_{\text{y}}=\text{B}{\text{E}}_{\text{y}}-\text{P}{\text{E}}_{\text{y}}, \end{array}$$

where  ER_y_ = emissions reductions in year y (tCO_2_e/yr), BE_y_ = baseline emissions in year y (*t*CO_2_e/yr), PE_y_ = project emissions in year y (tCO_2_e/yr).

(2) Data and parameters which are available at validation. Because the regional specific value is not available, the Intergovernmental Panel on Climate Change (IPCC) default value of the CO_2_ emission factor of coal was selected. Choose the CO_2_ emission factor corresponding to the applicable fuel type. IPCC default values may be used. CO_2_ emission factor of the fossil fuel used in the Project turbine prior to the implementation the Project (EF_FF,BL_) = 89.5 tCO_2_/TJ of fuel [12].

Use the latest version of approved “tool to determine the baseline efficiency of thermal or electric energy generation systems.” Depending upon the option selected from the latest version of approved “tool to determine the baseline efficiency of thermal or electric energy generation systems.” Energy efficiency of the turbine without retrofitting in a year y(*η*_BL,y_) = 39.553% (from Efficiency Test Report of the PAT prior to the retrofit).

(3) Ex-ante calculation of emission reductions. In order to estimate the emission reductions generated by the project, the following assumptions are brought into account. 

(4) Calculate the baseline emissions:

(6) EFBL,y=3.61000×EFFF,BL×FCPJ,y×NCVFF,PJηBL,y×HIPJ,y=3.61000×89.5×(322.5956  ×  2,632,000)×0.0293060.39553  ×  2,632,000  ×  8738.92=0.88126 tCO2eMMh,BEy=EGPJ,y∗EFBL,y=2,632,000∗0.88126=2,319,482 tCO2e.

(5) Calculate the project emissions:

(7) PEy=FCcoal,y×NCVcoal,y×EFCO2,coal,y=1,080,643.24 ton×0.023026 TJ/ton×89.5 tCO2e/TJ=2,227,019 tCO2e.

(6) Calculate the emission reductions:

(8) ERy=BEy−PEy=2,319,482−2,227,019=92,463 tCO2e.

#### 3.1.4. Summary of the Ex-Ante Estimation of Emission Reductions

It is expected that the project activities will generate emission reductions within the power grid for about 92,463 tCO_2_ *e* per annum over an 8-year fixed crediting period from 01/01/2012 to 31/12/2019. And the total emission reductions will come to 739,704 tCO_2_ *e*. 

### 3.2. Investment Analysis

#### 3.2.1. Determine the Suitable Financial Indicator for the Project Type

Financial indicator of internal rate of return (IRR) is the most suitable for such power retrofit project and decision making context. According to Trial Implementation Methods for Economic Assessment of technology retrofit Project in Power Engineering, the benchmark of IRR of power retrofit project is set at 8% [13]. The financial attractiveness of this project will be determined by comparing the IRR after tax (without Certified Emission Reductions (CERs)) with its benchmark applied in power retrofit project of China power industry, which is a standard recommended by the industry experts and is widely used at present in China. If the IRR after tax (without CERs) is less than 8%, the project is considered not being financially attractive in the absence of CDM revenues, and is therefore considered to be additional.

#### 3.2.2. Calculation and Comparison of Financial Indicators

The key parameters of the project are showed in Tables 2 and 3.

The financial analysis results are shown in Table 4. As shown in this table, without carbon credits, the IRR is −0.41%, which is much lower than the benchmark rate of 8%. This therefore indicates that in comparison to other alternative investments, the project without carbon credits is not financially attractive to a rational investor.

#### 3.2.3. Sensitivity Analysis

The sensitivity analysis is conducted to check whether, under reasonable variations of the sensitive factors in the critical assumptions, the results from the analysis remain unaltered. After overall checking of the IRR calculation sheet, five factors have been selected for the sensitivity analysis, which are the total investment, annual operation hours, electricity tariff, standard coal price, and the decrease of standard coal consumption.

Assuming the five factors within a fluctuation range from −20% to 20%, the IRR (after tax) of the project (without income from selling CERs) varies to a different extent, as shown in Table 5.

 As shown in the sensitivity analysis, even the varying range of the uncertain factors reaches ±20%, the IRR (after tax) could not reach the benchmark. The conclusion that the project is definitely not financially attractive would not be influenced. 

As above, the internal rate of return (IRR) of the project is only −0.41% without the revenue of carbon credits for example, CERs, which is much lower than the benchmark value of 8%. In addition, only when the unit price of carbon credit comes to 125 CNY/tCO_2_, the IRR could reach the benchmark and become financially feasible. Therefore the GHG emission reduction cost of such retrofit project is 125 CNY/tCO_2._

### 3.3. Design of Incentive Mechanism

#### 3.3.1. Incentive from Carbon Tax

Carbon tax is a Pigovian tax levied on the carbon content of fuels [14], which is a form of carbon pricing. Carbon is present in every hydrocarbon fuel (coal, petroleum, and natural gas) and is released as CO_2_, when they are burnt [15].

In this case study, it is assumed that government levies the carbon tax on coal consumption in the power sector. It means that the carbon tax will be a part of the fuel cost in operating the power plant, being of the same effect of raising the price of coal. According to the sensitive analysis, an effective carbon tax needs to increase the price of coal by 243 CNY/tce, so as to make the project financially feasible. Then the cost of carbon tax should be 90 CNY/tCO_2_ (1tce leads to GHG emission of 2.77 tCO_2_).

#### 3.3.2. Incentive from Carbon Market (Credit Trading)

Emissions trading or cap-and-trade is a market-based approach used to control pollution by providing economic incentives for achieving reductions in the emissions of pollutants. For GHG the largest is the European Union Emission Trading Scheme (ETS), whose purpose is to avoid dangerous climate change [16]. In the carbon market created by ETS, GHG emission reduction credits are a kind of eligible unit, generated from the project that is implemented by entity outside the ETS. As analyzed above, the price of carbon credits in the market for example, CERs must be not less than 125 CNY/tCO_2_ to make the project financially attractive.

#### 3.3.3. Incentives Combined with Encouraging Dispatch Plan

Since the electricity tariff is determined by the government in China, which is fixed unless the new tariff policy approved by the government, most of the coal fuel power plants resist carbon tax in practice. A very high cost of carbon tax is not very likely to occur in China. On the other hand, given the experience of the EU ETS, the credit price is of high volatility in the market [17]. Thus, there is always uncertainty about the credit price to make the investment decisions of this kind of retrofit. To mitigate the shortage of carbon tax and carbon market, the encouraging dispatch plan is expected to enhance the effect from above mechanisms.

Given the sensitivity analysis, the annual operation hours of the PAT are a very crucial factor to the IRR. The annual operation hours of a power plant are normally determined by power dispatch arrangement of grid company. In this section, the effect of encouraging dispatch plan is an analysis together with carbon tax and credit price in an ETS. Clearly, the more annual operation hours of the PAT, the higher IRR appears in the Project (Table 6). Thus, the necessary cost of either carbon tax and credit cost in ETS will decrease by incorporating with such encourage to dispatch plan.

## 4. Conclusions

This study presented the emission reductions of an ordinary thermal power plant after a steam turbine retrofit project with an incentive mechanism of carbon tax and carbon market (ETS). In particular, the most cost-efficient method is the combination of this mechanism made by central authorities with encouraging dispatch plan by grid company. The results presented within this paper indicate that the project will make a good demonstration for the other low efficiency thermal power plants in China.

## Figures and Tables

**Table 1:** The principal specifications of the steam turbine.

Item	Designed value
Manufacturer	Leningrad metal factory of Russia (designed in the beginning of 1970s)
Type of turbine	Supercritical, once reheated, single shaft, 4 cylinder and 4 steam exhaust, condensing turbine
Rated power	500 MW
Rated main steam flow	1528.8 t/h
Main steam pressure	23.54 MPa
Main steam temperature	540°C
Reheated steam pressure	3.51 MPa
Reheated steam temperature	540°C
Exhausted steam pressure	4.27/5.44 kPa
Number of blade stage	54
Net heat rate	8146 kJ/kWh
Lifetime	24 years

**Table 2:** The key parameters of the project.

Item	Value	Unit	Data source
Electricity supplied to the grid in year y (EG_PJ, y_)	2,632,000	MWh	Feasibility study report of the project (FSR)
Standard coal consumption after retrofit	322.5956	kg/MWh	Efficiency test report
Net calorific value of standard coal	29,306	MJ/ton	Efficiency test report
Net calorific value of fossil coal	23,026	MJ/ton	Calculated
Annual consumption of fossil coal	1,080,643.24	ton	Calculated
Average net heat consumption of turbine after retrofit	8738.92	kJ/kWh	Efficiency test report

**Table 3:** The key parameters of the proposed project.

Item	Value	Unit	Data source
Rated continuous power	500	MW	FSR
Total investment	100,000,000	RMB	FSR
Equity proportion	100	%	FSR
Incremental annual power generation	0	MWh	FSR
Profit loss due to the retrofitting	24,730,000	RMB	FSR
Annual operation hours	5600	h	FSR
Power consumption rate (for self-use)	6	%	FSR
Standard coal consumption for power generation before retrofit (designed in FSR)	316	g/KWh	FSR
Decrease of standard coal consumption after retrofit (designed in FSR)	13	g/KWh	FSR, P51
Standard coal price (without VAT)	389	RMB/ton	FSR
Electricity tariff (without VAT)	341.2	RMB/MWh	FSR
New added O and M cost	−11,675,000	RMB/year	FSR
Of which fuel cost saving due to retrofit	−14,175,000	RMB/year	FSR
New added repair cost	2,500,000	RMB/year	FSR
Income tax	25	%	FSR
Value added tax (VAT)	17	%	FSR
Town building maintenance tax	7 (of VAT)	%	FSR
Surcharge for education	3 (of VAT)	%	FSR
Project operating period	11	Years	FSR
Rate of residual value of the fixed assets	3 (out of total investment)	%	FSR
Depreciation period	11	Years	FSR
Amount of CERs	92,463	tCO_2_e/year	ER calculation sheet

**Table 4:** The financial indicators of the project.

Financial indicators	Rate
IRR (after tax) without CERs	−0.41%
Benchmark	8%
IRR with CERs	8.27%

**Table 5:** The sensitivity analysis of the project within a fluctuation range from −20% to 20%.

Fluctuation range	−20%	−10%	0	10%	20%
Total investment	2.34%	0.87%	−0.41%	−1.55%	−2.57%
Annual operation hours	−3.64%	−1.98%	−0.41%	1.08%	2.50%
Electricity tariff	−0.41%	−0.41%	−0.41%	−0.41%	−0.41%
Standard coal price	−3.64%	−1.98%	−0.41%	1.08%	2.50%
Decrease of standard coal consumption	−3.64%	−1.98%	−0.41%	1.08%	2.50%

**Table 6:** The necessary cost of carbon tax and credit price with/without encouraging dispatch plan.

Dispatch plan	Necessary cost of Carbon tax	Necessary cost of credit price
The dispatch electricity generation does not change	90 CNY/tCO_2_	125 CNY/tCO_2_
Dispatched electricity generation increased by 20%	50 CNY/tCO_2_	85 CNY/tCO_2_
